# Preparation and Optimization of an Ultraflexible Liposomal Gel for Lidocaine Transdermal Delivery

**DOI:** 10.3390/ma15144895

**Published:** 2022-07-14

**Authors:** Mengwei Sun, Ositomiwa O. Osipitan, Ewa K. Sulicz, Anthony J. Di Pasqua

**Affiliations:** 1 Department of Pharmaceutical Sciences, School of Pharmacy and Pharmaceutical Sciences, Binghamton University, 96 Corliss Ave., Johnson City, NY 13790, USA; msun22@binghamton.edu (M.S.); oosipit1@binghamton.edu (O.O.O.); esulicz1@binghamton.edu (E.K.S.); 2 Department of Biomedical Engineering, The Thomas J. Watson College of Engineering and Applied Science, Binghamton University, 4400 Vestal Pkwy. E., Binghamton, NY 13902, USA

**Keywords:** ultraflexible liposomes, transferosomes, lidocaine transdermal delivery, tail-flick test

## Abstract

The pain caused by lidocaine injections into the face prior to facial plastic surgeries intended to remove growths or tumorous lesions has been reported by many patients to be the worst part of these procedures. However, the lidocaine gels and creams currently on the market do not deliver an equal or better local anesthetic effect to replace these injections. To develop an alternative to the painful local anesthetic injection, we prepared ultraflexible liposomes using soy phosphatidylcholine, lidocaine, and different amounts of sodium cholate, a surfactant. The prepared ultraflexible liposomes (UFLs) were examined for particle size, zeta potential, cytotoxicity, and in vitro release. By using a carbomer as a gelling agent, the prepared UFL lidocaine gels were evaluated for their penetration ability in a Franz diffusion cell, using Strat-M membranes. The formulation achieving the highest amount of penetrated lidocaine was chosen for further pH, viscosity, and stability tests. The local anesthetic efficacy of the formulation was investigated by an in vivo tail-flick test in rats. Our findings suggested that this topical gel formulated with ultraflexible liposomal lidocaine has enhanced skin permeation ability, as well as an improved local analgesic effect from the lidocaine.

## 1. Introduction

When used as a local anesthetic, lidocaine functions via blocking the voltage-gated sodium channels, which induces a reversible block of action potential propagation [1]. Currently, lidocaine is mainly administered via subcutaneous, intramuscular, or intravenous injection, but this conventional delivery approach has many inherent limitations when used in the clinic. Anesthesia applied during surgical procedures dealing with superficial skin, such as skin transplantation, skin lesion removal, esthetic surgeries, tattooing, and birthmark/scar removal, usually demands numerous injections [2]. Multiple rounds of needle injections, as well as the invasive nature of injections, not only require administration by trained personnel but also elicit pain and discomfort, resulting in lower acceptance/compliance by patients [3,4,5]. The generation of sharp contaminants also poses safety problems [6]. Rationally, many issues involving injection could potentially be solved by advanced drug delivery methodologies, such as transdermal drug delivery. However, the available approaches for transdermal delivery usually require a long onset time. For example, the onset time for EMLA^TM^ cream, a commercially available topical anesthetic containing 2.5% lidocaine and 2.5% prilocaine, is 60 min, which might interfere with the efficiency of the surgery [2,7]. In addition, some dosage forms such as pastes, creams, and ointments for the transdermal delivery of drugs suffer from early removal caused by wetting, movement, and contact, which further necessitates that patients stay immobile. Thus, developing a biocompatible, bioadhesive, and efficient transdermal delivery vehicle, with a fast action time and prolonged anesthesia effect, is beneficial to both patients and physicians.

A variety of transdermal delivery methods have been developed for enhanced skin penetration, such as microparticles, ethosomes, solid lipid nanoparticles, and ultraflexible liposomes (UFLs) [8]. Among these systems, microparticles have bigger particle diameters and show less satisfactory skin permeation ability in comparison with solid lipid nanoparticles and UFLs, while ethosomes have the drawback of poor structural and chemical stability when in long-term storage, in addition to possible skin irritation caused by the high ethanol content [8]. Also known as (ultra)deformable liposomes, flexible liposomes, elastic liposomes, and transfersomes (first introduced by IDEA AG, Munich, Germany) [9], UFLs are biocompatible bilayer vesicles that can be loaded with various drugs to fulfill therapeutic, biochemical, and cosmetic purposes. They share some common features with traditional liposomes, but their formulation is optimized to achieve a more flexible and elastic lipid bilayer. Comprising a mixture of lipids with low phase transition temperatures and an appropriate amount of detergent, UFLs are often considered the vehicle of choice among dermal/transdermal delivery vehicles, largely due to their superior role as skin penetration enhancers, as well as their good long-term stability [10,11,12]. The detergent functions as a membrane destabilizer, leading to increased membrane deformability. The high deformability of UFLs has been reported to contribute to their skin penetration ability and can even enable drugs to reach systemic circulation [13,14,15].

The main drawback of UFLs when used topically is their liquidity, making it difficult to achieve localized application. To solve this issue, the nanoliposomes must be incorporated into an appropriate vehicle to protect the intrinsic structure of the vesicles. Carbomer has previously been proposed for sustained drug release and shows good bioadhesion; it has been used together with liposomes derived from soybean phosphatidylcholine [16,17]. Therefore, in this study, carbomer was used as a gelling agent for the incorporation of the UFLs and traditional liposomes (TLs) to develop a topical delivery system because of its low potential for skin irritation and sensitization, good bioadhesive properties, and good thermal stability.

To develop a noninvasive lidocaine delivery system to replace painful injections, first, we aimed to establish the ideal amount of surfactant needed for UFL lidocaine gel preparation to achieve the highest penetration ability. Then, careful characterization was performed to show the stability and viscosity profiles of the formulated gel. Finally, the analgesic effect was demonstrated through the tail-flick test in rats.

## 2. Materials and Methods

### 2.1. Materials

L-α-phosphatidylcholine (Soy) was purchased from Avanti Polar Lipids, Inc. (Alabaster, AL, USA). Chloroform, methanol, and Strat-M^®^ membranes were purchased from Millipore Sigma (St. Louis, MO, USA). Carbopol 940 and triethanolamine were purchased from Fisher Scientific (Hampton, NH, USA). Lidocaine was purchased from TCI AMERICA (Philadelphia, PA, USA). Dimethyl sulfoxide (DMSO) was purchased from VWR (Radnor, PA, USA). The MTS assay kit was purchased from Promega (Madison, WI, USA). Human keratinocytes were purchased from the American Type Culture Collection (ATCC) (Manassas, VA, USA).

### 2.2. Preparation of Lidocaine-Loaded (Ultraflexible) Liposomes

Lidocaine-loaded liposomal formulations were prepared using the thin-film hydration method [18]. First, 50 mg soybean phosphatidylcholine (SPC), 20 mg lidocaine, and different amounts (0, 10, 20, 30, and 50%, *w*/*w*) of sodium cholate (NaChol) were dissolved in a 2 mL methanol: chloroform mixture at a 1:1 *v/v* ratio (another formulation containing 35 mg SPC, 20 mg lidocaine, and 20% NaChol was prepared, using the same method). Using a rotary evaporator, a thin lipid film was formed on the internal surface of a round-bottomed flask. Then, the film was hydrated with 1 mL of PBS (pH = 7.4), followed by 5 min of sonication and vortexing. The resulting liposomal suspension was extruded through 200 nm polycarbonate membranes for particle homogenization.

### 2.3. Preparation of Liposomal Lidocaine Gel

To prepare the carbomer gel, 3.5 mg Carbopol 940 was added to 10 mL distilled water and stirred at 150 RCF in a refrigerator. At least 12 h later, triethanolamine was added slowly to the carbomer gel until the pH reached 7. Then, the carbomer gel was stirred using probe sonication at an amplitude of 20 MHz for 10 s, followed by 1 min of vortexing. To prepare liposomal lidocaine gel, liposomal suspension (or lidocaine solution) and carbomer gel in a 3:1 *v/v* ratio was mixed by vortexing and stirring for 5 min. For a follow-up experiment where DMSO was tested as a chemical enhancer, 3% DMSO was added during the mixing of the liposomal suspension and carbomer gel.

### 2.4. Permeation Studies

Transdermal diffusion assays were conducted using a Franz cell and Millipore Strat-M membranes, a synthetic membrane model suitable for predicting transdermal diffusion in human skin. The Franz diffusion cells were loaded with 50 mg samples. The receptor compartment consisted of a mixed media containing PBS (pH = 7.4) and 20% ethanol. Ethanol was added to better solubilize the lidocaine released in the media. All experiments were carried out for 3 h at 32 ± 0.5 °C, with constant stirring at 200 RCF. Before use, the Strat-M membrane was pre-hydrated for 1 h with the release media and was then mounted on the top of the receptor chamber of the Franz cell, with an effective diffusion area of 1.76 cm^2^. Then, 50 mg of various formulations of lidocaine was applied onto the membrane surface and spread evenly, to cover all the exposed area of the membrane. Aliquots of the receptor solution were collected at various time points and were replaced with the same volume of release media. The lidocaine concentration was read spectrophotometrically at λ = 263 nm. Permeation studies were also performed by loading 200 mg samples onto the membrane using the same technique. The permeation of each formulation was conducted in triplicate; the results were shown as the mean ± SD. Due to the findings in the permeation studies, an ultraflexible liposomal formulation prepared by SPC and 20% NaChol was chosen for further examination. In the following text, the term “ultraflexible” refers to this formulation if there is no specific alternative explanation.

### 2.5. Characterization of UFL and TL Nanoparticles Loaded with Lidocaine

First, 900 µL of deionized water was added to 100 µL of UFL or TL suspensions, to investigate the zeta potential and the particle size of the liposomes. The zeta potential and particle size were measured via the technique of dynamic light scattering, using a Malvern Zetasizer (Nano ZS ZEN 3600; Malvern Instruments, Malvern, Worcestershire, UK).

### 2.6. In Vitro Release Study

The in vitro release study was performed using the dialysis method. Briefly, an equivalent amount of 1 mL of lidocaine-loaded UFL or TL dispersion was introduced into dialysis bags with a molecular weight cutoff of 12,000 kDa. The dialysis bags were suspended in a release media containing PBS and 20% ethanol at 37 °C, at a speed of rotation of 200 RCF, and placed within a closed glass vial. The samples (500 μL) were withdrawn and analyzed spectrophotometrically at λ = 263 nm at predetermined time points. The withdrawn samples were replaced with the same volume of PBS. The release of lidocaine from the liposomal formulations was compared to the first-order kinetic model using the following equation:

(1) $$\frac{Q}{Q_{0}}=1-e(-k\times t)$$

where *Q* represents the amount of drug released at time *t*; *Q*_0_ is the initial amount of drug; *k* is the release constant.

### 2.7. Cytotoxicity Assays

Human keratinocytes were cultured to indicate the cytotoxicity of the UFLs and TLs that were prepared. Keratinocytes were seeded in a 96-well plate at a density of 5000 cells/well; each well contained 100 μL of culture media. After 24 h, the culture media was replaced with 10 µL of treatment containing either UFLs or TLs (or PBS as a negative control) and 90 µL of fresh complete growth media, so that the cells were treated with 8.75 mg/mL UFL or 7 mg/mL TL. After treating the cells for 24 h, the old media with the treatment was discarded, and fresh media containing MTS solution was added. The UV-Vis absorbance of the cells was read at 490 nm after 2 h of incubation.

### 2.8. Viscosity Tests

The viscosities of the formulations were determined using a microVISC from RheoSense (San Ramon, CA, USA) at a skin temperature of 32 °C. Viscosity assays were performed for ULF and TL lidocaine gels and a commercially available product, LMX4 cream. Briefly, 10 μL of the sample was loaded into the viscometer for each viscosity measurement, and the viscosity was determined at shear rates of 10 to 60 s^−1^ at 32 °C. Samples were run in triplicate.

### 2.9. Homogeneity of the UFL Lidocaine Gel

The lidocaine homogeneity of the UFL lidocaine gel was investigated to ensure uniform dispersion or distribution of the lidocaine within the UFL gel. To examine the lidocaine content uniformity, samples collected from three different areas of the gel were mixed with a sufficient quantity of Triton X-100 to break the lipid membranes and release the drug. Then, the lidocaine concentration of the samples was analyzed spectrophotometrically at λ = 263 nm. Three batches of the UFL lidocaine gel were used for this experiment. Drug content uniformity percentages were calculated, using the following equation:

(2) $$\frac{\text{Lidocaine in sample (mg)}}{\text{Mean lidocaine content (mg)}}\times 100\%.$$

### 2.10. Stability of the UFL Lidocaine Gel

Stability tests were used to examine whether lidocaine will leak from the UFL gel when in long-term storage. The prepared UFL lidocaine gel formulations were kept in a tightly closed glass vial at 4 °C for 90 days. The lidocaine concentration in the UFL gel that was stored for 0, 30, 60, and 90 days was determined spectrophometrically at λ = 263 nm. The appearance of the stored gel preparations was also recorded periodically.

### 2.11. Tail-Flick Test

The experimental animal protocol for this study was approved by the Binghamton University Institutional Animal Care and Use Committee. For the tail-flick assay, 20 male Sprague Dawley rats weighing 290 ± 25 g were distributed into four groups (*n* = 5) with 5 extra rats. All rats were housed under specified environmental conditions (temperature: 20 ± 2 °C, humidity: 40 ± 10% RH) throughout the study. The rats were accommodated to the above-mentioned conditions for 10 days prior to the tail-flick assay. This experiment used a tail-flick meter manufactured by Data Sciences International (St. Paul, MN, USA). During the experiment, a predetermined specific area of rat tail was placed under a radiant heat source, while the rats were kept immobile. Before testing any samples on the rats, the baseline latency for every rat was tested, which was determined as the average time across three measurements. The rats whose baseline latency average time was not in the time range of 6–8 s were ruled out from the study. A 20-s cutoff time was set on the tail-flick meter to avoid tissue injury caused by overheating. The tail-flick latency time was recorded as the duration from the start of heat exposure to the occurrence of the flicking of the tail. The four groups of the rats were treated with 50 mg carbomer gel (first group), 50 mg UFL lidocaine gel (second group), 50 mg TL lidocaine gel (third group), and 50 mg LMX4 cream (fourth group). The time course of the anesthetic effect of each formulation was shown by plotting the average of the latency times as a function of time.

### 2.12. Statistical Analysis

All *p*-values were calculated using the Microsoft Excel *t*-test (a two-sample test assuming unequal variances) function (Redmond, WA, USA).

## 3. Results

### 3.1. Optimization of UFL Formulation through Permeation Studies

**Figure 1:**
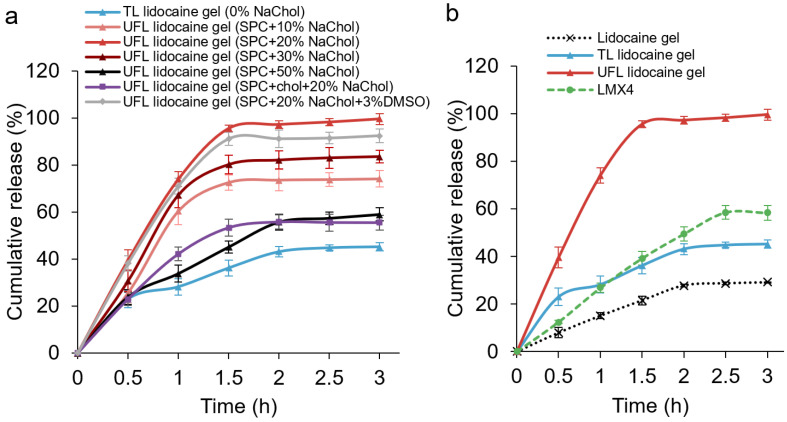
Permeation profiles through Strat-M membranes of (**a**) 50 mg different formulations of liposomal gels and (**b**) lidocaine-containing carbomer gel, LMX4 cream, TL and UFL lidocaine gels.

A transdermal diffusion study was conducted using a Franz cell to test the gels’ skin permeation ability. First, we examined the penetration ability of different formulations of liposomes with various percentages of the surfactant, NaChol. It can be seen from Figure 1a that the amount of lidocaine that penetrated through the Strat-M membrane increased when the NaChol percentage increased from 0 to 20% and decreased when the NaChol kept increasing from 20% to 50%. Having determined that 20% NaChol was the ideal amount of surfactant for the SPC liposomes, we tried adding cholesterol when preparing the liposomes, or including DMSO during liposomal gel formation, to see if cholesterol or DMSO would further enhance the drug penetration. Unfortunately, neither of these two factors demonstrated a better release profile than liposomes made from SPC with 20% NaChol. Therefore, this optimal formulation was compared to plain lidocaine gel and LMX4 cream (Figure 1b). Almost all the lidocaine (95.52%) that was encapsulated in the UF liposomal lidocaine gel penetrated through the skin within the first 1.5 h, while the TL lidocaine gel and LMX4 cream demonstrated a 36.17% and 39.2% cumulative release of lidocaine, respectively. From these permeation studies, it can be concluded that adding 20% NaChol to the liposomal formulation greatly improved the skin penetration ability, making the ultraflexible liposomal vehicle an effective tool in transdermal drug delivery.

### 3.2. Characterization of UFLs and TLs

In light of the results from the above permeation studies, UFLs prepared using SPC with 20% NaChol were chosen for further studies; “UFL” in the following text refers to this specific formulation. We characterized the size, PDI, and surface potential of this UFL formulation and the TL formulation before incorporating them into gels. The Zetasizer data of UFLs and TLs (Table 1) showed that both formulations had uniform size distribution, as indicated by their PDIs. Even when loaded with lidocaine, both formulations showed a small particle size, since the interaction between the liposome and drug is negligible [19]. When containing a surfactant in the formulation, UFLs had a smaller average size and a negative surface charge compared to TLs, indicating that UFLs are more stable and have a more favorable size distribution [2].

### 3.3. In Vitro Release Profiles and Cytotoxicity of UFL and TL Dispersions

**Figure 2:**
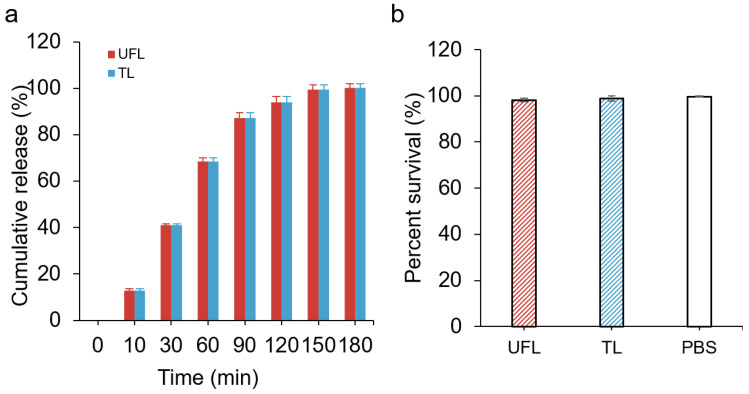
(**a**) Release profiles of UFLs and TLs; (**b**) percentage survival of human keratinocytes treated with UFL, TL, or PBS (negative control) for 24 h.

The dialysis release profiles of lidocaine of the prepared UFLs (prepared by SPC with 20% NaChol) and TLs are demonstrated in Figure 2a. More than 80% of the lidocaine content from the prepared UFLs, as well as the TLs, was released after 90 min. Statistical analysis confirmed that there is no significant difference in the release percentages of lidocaine from these two formulations (*p* > 0.05). Both formulations released their drug contents based on a first-order kinetic model (R^2^ > 0.95). The controlled release of lidocaine from UFLs and TLs, as shown in the figure, was expected due to the role of liposomes as a drug reservoir for prolonged release. Both UFLs and TLs showed good biocompatibility. A colorimetric MTS assay was performed to test the cytotoxicity of these liposomal formulations in human keratinocytes. The cells were treated with 10 µL of non-diluted UFL (prepared by SPC with 20% NaChol) or a TL suspension plus 90 µL of culture media. As shown in Figure 2b, the percentage survival rates of these liposomes were both greater than 98%, indicating the great biocompatibility of SPC liposomes.

### 3.4. The Uniformity, Viscosity, and Stability of UFL Lidocaine Gel

**Figure 3:**
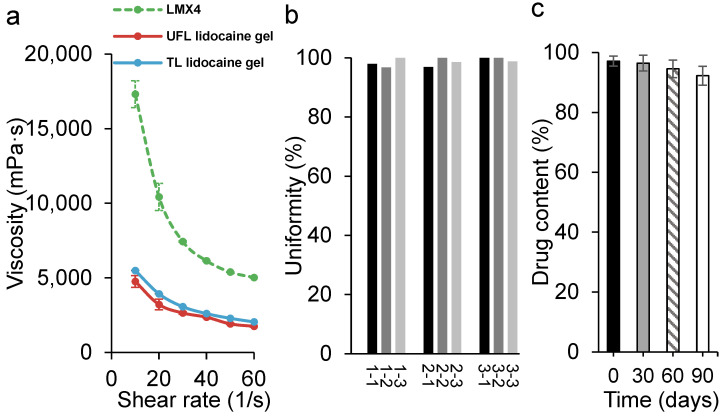
(**a**) The viscosity of LMX4 cream (green line) and the UFL (red line) and TL (blue line) lidocaine gels at 32 °C; (**b**) lidocaine content uniformity of the UFL lidocaine gel (three samples were taken from each batch of the gel; three batches were included); (**c**) stability of the UFL lidocaine gel over 3 months’ storage at 4 °C.

The viscosity of LMX4 cream, UFL, and TL lidocaine gels was examined at 32 °C, to mimic the skin surface temperature. As indicated in Figure 3a, even though liposomal lidocaine gel groups showed a lower viscosity overall compared to the other two commercial products, they both followed the same trend; as the shear rate increased, their viscosity decreased. In terms of the drug content uniformity of the UFL lidocaine gel, insignificant changes (*p* = 0.63) in the lidocaine content were detected, showing the good homogeneity of the prepared gel. The UFL lidocaine gel also exhibited excellent stability since no significant decrease (*p* = 0.19) in drug content was observed over 3 months for the stored formulations at 4 °C (Figure 3c).

### 3.5. Tail-Flick Test for Local Anesthetic Action

**Figure 4:**
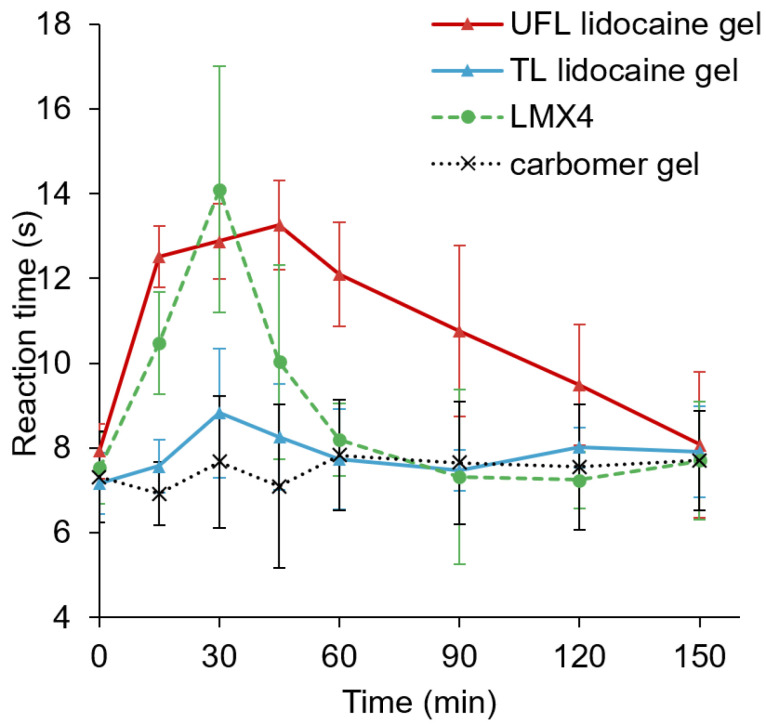
The anesthetic effect of carbomer gel (negative control), TL lidocaine gel, UFL lidocaine gel, and LMX4 cream on the latency of tail-flick after 50 mg of lidocaine-equivalent application.

The tail-flick test is a test of the pain response in animals, which is used in basic pain research to demonstrate the effectiveness of analgesics through the observation of the animals’ reaction to heat. In this test, these rodents’ tails are given different topical lidocaine products that are meant to weaken their reactions to pain. As a result of these weakened responses to pain, we can test the effect of the drugs by exposing the tail to constant heat and recording how long it takes to flick, representing the rat’s response to the pain. Figure 4 shows the reaction time taken by the rats to flip their tails away from the heat stimulus after the application of different lidocaine-containing formulations or plain carbomer gel (negative control). An increased reaction time was observed in both the UFL lidocaine gel and LMX4 groups, after applying the formulations on the tails for 15 min. However, the analgesic effect plateaued after 30 min of application in the LMX4 group, while the UF group maintained a prolonged effect after 2 h. This difference can also be noted in the values of the area under the curve (AUC). The maximum AUC_0__–150min_ value was calculated to be 1639.97 ± 201.64 s minutes (s·min) for the UFL lidocaine gel group, which is significantly higher (*p* < 0.01) compared to the control group (1127.79 ± 206.61 s·min), significantly higher (*p* = 0.02) compared to the LMX4 group (1311.86 ± 224.41 s·min), and significantly higher (*p* < 0.01) compared to the TL lidocaine gel group (1180.76 ± 127.76 s·min).

## 4. Discussion

Treating pain properly in the clinic is still a challenge, and the current analgesics and nonpharmacologic medications available present limited options for healthcare professionals [20]. The toxic effects of over-the-counter and prescription systemic therapies, including acetaminophen, non-steroidal anti-inflammatory drugs (NSAIDs), and opioids have been reported and usually result in dose-limiting adverse effects [21,22,23,24,25]. Interest in and the use of topical anesthetic agents has been growing, possibly owing to their potential application in acute and chronic pain relief, as well as their relative lack of side effects. One topical drug that has been commonly applied in the clinic is the local anesthetic, lidocaine. Lidocaine is efficacious both systemically and topically, and it can be found in various prescriptions and over-the-counter formulations, including gels, creams and ointments, sprays, and patches [20].

For topical lidocaine formulations to function, the lidocaine must penetrate the outer layer of the skin, the stratum corneum (SC), which acts as a natural barrier. Loading water-soluble lidocaine in liposomes is a relatively simple and effective method for achieving this penetration. Liposomes can travel more easily through the epidermal layer compared to other conventional dosage forms because their lipid composition resembles the epidermis. However, most topically applied TLs accumulate in the upper layers of the SC and act as a “reservoir”, leading to a more localized effect [26].

In order to further improve the skin permeation of the loaded drug, changes in the composition and structure of TLs were made to create new types of lipid vesicles with flexible and ultra-deformable features, including UFLs. Numerous reports have indicated that UFLs are superior to TLs in terms of lidocaine transdermal delivery [2,27,28]. UFLs possess a highly deformable bilayer and have increased water binding and retaining capacity [29,30]. In the case of nonocclusive skin application, they are able to reach the deeper strata (water-rich portion) in skin tissue, contributing to the spontaneous transport of the drug-encapsulated vehicles through the skin barrier [29,31,32]. In this study, we chose not to separate the unencapsulated/free drug from the liposomes, since it has been reported that liposomes not only affect the penetration of the encapsulated drugs but also the penetration of the non-entrapped drug [12,33,34]. When the liposomes suspension interacts with the skin, all the components are in contact with the SC; the fluidization of the lipid membranes in the skin that are generated by the components of the liposomes improves the transdermal penetration of all drug molecules, including those trapped in the liposomal bilayer or the aqueous core, as well as the free drug molecules in the aqueous buffer on the exterior of the liposomes [12].

The lipid-to-surfactant ratio significantly influences the flexibility of the bilayer of UFL vesicles [31]. We optimized the UFL formulation by adjusting the amount of the surfactant, NaChol, and adding cholesterol or DMSO; then, we tested the penetration abilities of the prepared formulations in the carbomer gel system, using synthetic Strat-M membranes (Figure 1). The Strat-M membrane has been shown to have similar morphological features to human skin, such as thickness, pore size, surface morphology, and diameter [35]. Moreover, the similarities of the Strat-M membrane to human skin have also been demonstrated in numerous permeation studies [35,36,37,38]. Therefore, Strat-M can be used as an effective alternative to human skin when carrying out transdermal permeation experiments. Our results, conducted using Strat-M membranes, showed that all the tested UFL preparations deliver significantly more lidocaine in 3 h than the TL formulation, proving the enhanced skin penetration effect of UFLs using NaChol as a surfactant. Among the various NaChol percentages in the UFL formulations, we found that 20% NaChol achieved the highest penetrated lidocaine level. Since DMSO has been used as a penetration enhancer [39] and cholesterol has also been included in the liposomal constitution, together with SPC, in terms of transdermal delivery [2], we added DMSO or cholesterol to the 20% NaChol formulation. No significant improvement was observed for the DMSO-including formulation, while the cholesterol-including formulation resulted in a significantly decreased amount of penetrated lidocaine (Figure 1). This is probably because cholesterol is positively correlated with the fluidity, permeability, membrane strength, elasticity, and stiffness of the lipid vesicles [40], thus resulting in weakened performance in terms of the transdermal delivery of lidocaine.

After the determination of an optimized UFL formulation, numerous physical characterization and cytotoxicity tests of this formulation were conducted and the results were compared to the TL formulation. DLS was performed for the lipid suspension samples of UFL and TL, before incorporating them into the gels. UFLs showed a more suitable size distribution compared to TLs (Table 1), since vesicles with a size of less than 300 nm are capable of transporting their loaded drug into the deeper layers of the skin, to a certain extent; however, those vesicles with a size of less than 70 nm have shown maximum drug delivery in both the viable epidermis and dermis [33]. This might contribute to the enhanced skin permeation outcome of UFLs as mentioned above. To examine the toxicity of UFLs and TLs, human keratinocytes were exposed to UFL or TL formulations at a range of concentrations for 24 h (Figure 2b). Our results showed that there was no toxicity toward keratinocytes, even when treated with 10% UFL (8.75 mg/mL) or TL (7 mg/mL) suspensions, which indicated the good biocompatibility of SPC and NaChol and provided a safe basis for further in vivo tests.

After incorporating the UFL and TL suspensions into gels, we examined the viscosity and stability of these gels. The results from the viscometer (Figure 3a) exhibited non-Newtonian behavior since a higher shear rate induced a decrease in viscosity, which is preferable in topical semisolid formulations [41]. Non-Newtonian formulations are easy to spread onto the skin but do retain a level of solidity when at rest, such as in the container. High viscosity when at rest also makes the formulation less likely to be prematurely removed from the skin after being applied. The drug content uniformity and stability of the UFL gel were confirmed in Figure 3b,c, indicating that encapsulating UFLs into a gel is beneficial for the stability of UFLs because the increased viscosity of the system may reduce the possibility of infusion [27]. These characteristics of the UFL preparation verified that 20% of NaChol was suitable for the carbomer gel system.

The in vivo anesthetic study was conducted to investigate the effectiveness of UFLs as lidocaine carriers. As shown in Figure 4, the UFL gel group presented a faster onset, as well as a more prolonged anesthetic effect, compared to the TL gel group, suggesting that UFLs facilitated the transdermal permeation of lidocaine and prolonged the anesthetic duration. In addition, the characteristics of biocompatibility and colloidal stability of UFLs may contribute to their prolonged anesthetic effect [2]. The protective role of UFLs against lidocaine metabolization is also a possible influencing factor for their long-term effect [42].

In this study, we compared the UFL gel to LMX4 in multiple experiments. LMX4 is a lipid-based lidocaine cream that is sold over the counter; it has been used extensively in clinical studies to compare with prescription-only medications such as EMLA. LMX4 was suggested to be an equally effective alternative topical analgesic to EMLA cream for newborn male circumcision, and LMX4 might offer an improved risk-benefit profile due to the presumed faster onset of action, with no risk of methemoglobinemia [43]. In a more recent clinical trial, LMX4^®^ was preferred by the patients as their choice of topical anesthetic for dermatological laser and skin micro-needling procedures among EMLA, Ametop™ (4% tetracaine gel), and LMX4 [44]. Based on our results, UFL gel achieved enhanced penetration ability and anesthetic effect in both in vitro and in vivo tests compared to LMX4; therefore, UFL gel may be considered a potential alternative to the current topical anesthetics in a wide range of applications.

## 5. Conclusions

Liposomes are one of the most common drug carriers used in a variety of fields. Embedding the liposomal suspension in gel dosage form not only provides the possibility of localized skin application but also increases the stability of liposomes. With the help of the detergent NaChol in the UFL gel, improved skin permeation, as well as an enhanced local anesthetic effect, is achieved. Compared to the commercially available lipid-based lidocaine cream, LMX4, UFLs possess a better in vitro skin penetration profile and enhanced anesthetic ability in vivo, with a longer duration of action. Considering that LMX4 has been reported as effective analgesia for newborn circumcision, and the most preferred topical anesthetic for dermatological laser treatment and skin micro-needling, it can be concluded that UFL gels may be useful for the development of an effective alternative to painful lidocaine injections and the current topical lidocaine dosage forms in clinical settings.

## Figures and Tables

**Table 1:** The size, PDI, and zeta potential of UFLs and TLs.

Formulations	Size (nm)	PDI	Zeta Potential (mV)
UFL	64.3 ± 2.1	0.08 ± 0.01	−21.6 ± 1.2
TL	139.3 ± 1.8	0.09 ± 0.01	0

## Data Availability

The data presented in this study are available on request from the corresponding author.

## Abbreviations

UFL	Ultraflexible liposome
TL	Traditional liposome
SPC	Soybean phosphatidylcholine
NaChol	Sodium cholate
NSAIDs	Non-steroidal anti-inflammatory drugs
